# Macrofilaricidal Activity after Doxycycline Only Treatment of *Onchocerca volvulus* in an Area of *Loa loa* Co-Endemicity: A Randomized Controlled Trial

**DOI:** 10.1371/journal.pntd.0000660

**Published:** 2010-04-13

**Authors:** Joseph D. Turner, Nicholas Tendongfor, Mathias Esum, Kelly L. Johnston, R. Stuart Langley, Louise Ford, Brian Faragher, Sabine Specht, Sabine Mand, Achim Hoerauf, Peter Enyong, Samuel Wanji, Mark J. Taylor

**Affiliations:** 1 Filariasis Research Laboratory, Molecular and Biochemical Parasitology, Liverpool School of Tropical Medicine, Liverpool, United Kingdom; 2 Department of Life Sciences, Faculty of Science, University of Buea, Buea, Cameroon; 3 Research Foundation in Tropical Diseases and Environment (REFOTDE), Buea, Cameroon; 4 Institute of Medical Microbiology, Immunology and Parasitology (IMMIP), University Hospital Bonn, Bonn, Germany; 5 Tropical Medicine Research Station, Kumba, Cameroon; Centers for Disease Control and Prevention, United States of America

## Abstract

**Background:**

The risk of severe adverse events following treatment of onchocerciasis with ivermectin in areas co-endemic with loiasis currently compromises the development of control programmes and the treatment of co-infected individuals. We therefore assessed whether doxycycline treatment could be used without subsequent ivermectin administration to effectively deliver sustained effects on *Onchocerca volvulus* microfilaridermia and adult viability. Furthermore we assessed the safety of doxycycline treatment prior to ivermectin administration in a subset of onchocerciasis individuals co-infected with low to moderate intensities of *Loa loa* microfilaraemia.

**Methods:**

A double-blind, randomized, field trial was conducted of 6 weeks of doxycycline (200 mg/day) alone, doxycycline in combination with ivermectin (150 µg/kg) at +4 months or placebo matching doxycycline + ivermectin at +4 months in 150 individuals infected with *Onchocerca volvulus*. A further 22 individuals infected with *O. volvulus* and low to moderate intensities of *Loa loa* infection were administered with a course of 6 weeks doxycycline with ivermectin at +4 months. Treatment efficacy was determined at 4, 12 and 21 months after the start of doxycycline treatment together with the frequency and severity of adverse events.

**Results:**

One hundred and four (60.5%) participants completed all treatment allocations and follow up assessments over the 21-month trial period. At 12 months, doxycycline/ivermectin treated individuals had lower levels of microfilaridermia and higher frequency of amicrofilaridermia compared with ivermectin or doxycycline only groups. At 21 months, microfilaridermia in doxycycline/ivermectin and doxycycline only groups was significantly reduced compared to the ivermectin only group. 89% of the doxycycline/ivermectin group and 67% of the doxycycline only group were amicrofilaridermic, compared with 21% in the ivermectin only group. *O. volvulus* from doxycycline groups were depleted of *Wolbachia* and all embryonic stages *in utero*. Notably, the viability of female adult worms was significantly reduced in doxycycline treated groups and the macrofilaricidal and sterilising activity was unaffected by the addition of ivermectin. Treatment with doxycycline was well tolerated and the incidence of adverse event to doxycycline or ivermectin did not significantly deviate between treatment groups.

**Conclusions:**

A six-week course of doxycycline delivers macrofilaricidal and sterilizing activities, which is not dependent upon co-administration of ivermectin. Doxycycline is well tolerated in patients co-infected with moderate intensities of *L. loa* microfilariae. Therefore, further trials are warranted to assess the safety and efficacy of doxycycline-based interventions to treat onchocerciasis in individuals at risk of serious adverse reactions to standard treatments due to the co-occurrence of high intensities of *L. loa* parasitaemias. The development of an anti-wolbachial treatment regime compatible with MDA control programmes could offer an alternative to the control of onchocerciasis in areas of co-endemicity with loiasis and at risk of severe adverse reactions to ivermectin.

**Trial Registration:**

Controlled-Trials.com ISRCTN48118452

## Author Summary

The control of onchocerciasis in Africa relies on the sustained delivery of ivermectin. In certain areas, annual treatments delivered with high population coverage for at least 15–17 years can break transmission. In other endemic settings this strategy alone is thought to be insufficient to eradicate the disease. One of the major limitations occurs in areas that are co-endemic with another filarial infection caused by *Loa loa*, due to the risk of a rare severe adverse event associated with the rapid killing of *L. loa* microfilariae in heavily parasitized individuals. There are also concerns over recent evidence of reduced efficacy of ivermectin and the possible development of resistance. An alternative approach is to target the *Wolbachia* bacterial endosymbionts of *Onchocerca volvulus* with the antibiotic, doxycycline. In an area of Cameroon co-endemic for onchocerciasis and loiasis we conducted a trial comparing doxycycline with or without ivermectin treatment to ivermectin treatment alone. A six-week course of doxycycline delivers macrofilaricidal and sterilizing activities, which is not dependent upon co-administration of ivermectin. Doxycycline is well tolerated in patients co-infected with moderate intensities of *L. loa* microfilariae. The trial indicates that anti-wolbachial therapy is a feasible alternative to ivermectin in communities co-endemic for onchocerciasis and loiasis.

## Introduction

Onchocerciasis (also known as River Blindness) is a chronic disease induced by the filarial nematode *Onchocerca volvulus*. An estimated 37 million individuals are infected worldwide with 90 million at risk of infection, mainly in Sub-Saharan Africa. Adult worm infections establish within subcutaneous nodules (onchocercomas) and produce microfilariae (mf), which parasitize skin and eye tissues. Mf are the transmissive stage for black fly vectors and are also responsible for the major disease pathologies of onchocerciasis, including intense troublesome itching, dermatitis, atrophy, visual impairment and blindness.

Currently, the only drug available to treat onchocerciasis is ivermectin (Mectizan^TM^, Merck). Ivermectin is generally a safe and effective microfilaricide and has been used successfully in community-directed treatment programs aimed at both reducing the burden of disease and controlling transmission since 1987 [1],[2]. Ivermectin has some macrofilaricidal activity against female adult worms after 6 years of exposure [3], or when given repeatedly at three-monthly intervals [4], [5]. Higher doses of ivermectin do not improve on this activity and such regimens are contraindicated due to the occurrence of visual problems [6]. Another anti-filarial drug, diethylcarbamazine (DEC), is also contraindicated due to the incidence of treatment-associated blindness and the frequent development of potentially life threatening adverse reactions, known as Mazzotti Reactions [7], [8].

There are three major limitations of a sole reliance on ivermectin for onchocerciasis control. Firstly, its use in areas co-endemic with *Loa loa*, a tissue dwelling filariae that gives rise to blood circulating mf and found principally in forested regions in Africa. Reports of severe adverse reactions (SAE), including encephalopathy, coma and death, in the Central Africa region following mass distribution of ivermectin have introduced serious concerns and disruptions to onchocerciasis control programs [8]. Although the mechanism of ivermectin-associated SAE has not been fully elucidated, *L. loa* mf have been detected in the cerebral spinal fluid of patients suffering severe adverse reactions, indicating that mf can cross the blood brain barrier. The intensity of *L. loa* mf in the blood has been determined to be a major risk factor in the development of SAE [8].

Secondly, because ivermectin principally targets the mf stage, continuous delivery of annual treatment is required for at least 15–17 years to interrupt transmission as demonstrated in some endemic areas of Africa [9]. In other endemic areas of Africa this strategy is unlikely to lead to the interruption of transmission due in part to civil strife and conflict, insufficient health infrastructure and political commitment to funding for sustained control programmes, which together compromise the eradicability of onchocerciasis in Africa [10].

The third limitation is that such a long term, community-based strategy based on a single drug intervention is potentially vulnerable to the development of drug resistance. Recent reports from Ghana show evidence of sub-optimal efficacy of ivermectin in communities receiving 6–18 rounds of treatment [11], [12], [13]. Parasites from these communities show genetic changes associated with resistance to ivermectin in other nematodes and increase the concern of resistance to ivermectin developing in onchocerciasis [14], [15].

Considering the absence of any safe alternative to ivermectin, there is an urgent need to identify novel anti-filarial drugs. An ideal alternative would exhibit curative (macrofilaricidal) or permanent sterility and have minimal treatment-associated side effects and be safe to use in patients co-infected with *L. loa*.

A promising approach is to use antibiotics such as doxycycline to target not the filariae itself, but the *Wolbachia* endosymbiotic bacterium that is found in all life stages of *O. volvulus*. Pilot, open-labelled trials in onchocerciasis have demonstrated that 6-week courses of 100 mg/day oral doxycycline cause >90% reductions in *Wolbachia* levels from filarial tissues followed by an almost complete and sustained absence (12–18 months) of mf from the skin [16], [17], [18]. Deleterious effects on embryogenesis were determined by histological assessment of extirpated nodules. However, a clear adulticidal effect of doxycycline could not be determined in onchocerciasis patients after 18 months [18]. More recent placebo controlled trials with extended follow-up analysis have detected significant macrofilaricidal activity 21–27 months after receiving 4 to 6 week courses of 200 mg doxycycline [19] or a 5-week course of 100 mg doxycycline [20].

As *L. loa* is free of *Wolbachia* symbionts [21], [22] antibiotic therapy is not an option for their treatment. This, however, could be an advantage for the treatment of concomitant onchocerciasis or lymphatic filariasis with antibiotics in individuals co-infected with *L. loa* without the risk of microfilaricidal induced SAE.

We therefore carried out a randomized, double-blind, placebo controlled field trial to assess the efficacy and safety of a six-week course of 200 mg/day oral doxycycline with or without ivermectin for the treatment of onchocerciasis alone and in patients co-infected with *L. loa*. A proportion of the onchocerciasis patients were also co-infected with *Mansonella perstans*. Our primary objectives were to measure changes in a) *O. volvulus* mf levels in the skin, b) *Wolbachia* levels in adult *O. volvulus* tissues, c) embryogenesis within female *O. volvulus* uteri and d) adult motility and viability. Secondary objectives were to measure the incidence and severity of adverse events and changes in *L. loa* and *M. perstans* microfilaraemia.

## Methods

### Ethics statement and trial registration

The experimental protocol for this study was designed in accordance with the general ethical principles outlined in the Declaration of Helsinki. The trial was approved by ethics committees of the Tropical Medicine Research Station, Kumba and the Research Ethics Committee of The Liverpool School of Tropical Medicine. Written informed consent was obtained from all participants, with the exception of those who were illiterate, where a literate witness signed on behalf of the participant and the participant added a thumbprint. The trial is registered with the current controlled trials registry, no: ISRCTN48118452.

### Participants

The trial was community based and was undertaken in 6 satellite villages (Bifang, Ebendi, Eka, Ngalla, Dinku and Olurunti) of the market town of Widikum, in the North West Province of Cameroon (between latitude 5° N 43–5° N 54 and between longitude 9° E 41–9° E 44) starting on 1^st^ July 2003 and finishing on 31^st^ March 2005. The area is hyperendemic for onchocerciasis with a community prevalence of *Loa loa* ranging from 3.36%–14.29% [23]. Nodulectomy surgery was performed at Batibo Hospital under the direction of The District Health Officer. Individuals eligible for participation were adults of both sexes aged 15–60, with a minimum body weight of >/ = 40 Kg, in good health without any clinical condition requiring chronic medication. Mf counts were assessed microscopically following skin biopsy using a Walser skin punch. Hepatic and renal function and pregnancy were assessed by dipstick chemistry. Exclusion criteria encompassed an *O. volvulus* microfilarial load <10 mf/mg, a *L. loa* microfilarial load >8000 mf/ml, hepatic and renal enzymes outside of normal ranges (AST [0–40 IU/l, ALT [0–45 IU/l] and creatinine [3–126 µmol/l]) pregnancy, lactation, intolerance to ivermectin, alcohol or drug abuse or anti-filarial therapy in the last 12 months.

### Intervention

Participants received 2×100 mg capsules of doxycycline (Vibramycin^TM^, Pfizer) or matching placebo supplied by the manufacturer, daily, for a total of 42 days following a breakfast meal. Four months after the start of treatment, participants received an oral dose of 150 µg/kg ivermectin (Mectizan^TM^, Merck & Co. Inc.) or dummy pill (non-matching lactose tablet). Treatment was delivered by trained community distributors who gave the drug/placebo to the participants and witnessed them swallowing the tablets.

### Outcomes

Outcome measurements encompassed: a) the number of mf present in skin snip biopsies taken at baseline, 4, 12 and 21 months after the start of treatment, b) the quantity of a *Wolbachia* single copy gene (*Wolbachia* surface protein; *wsp*) within extirpated nodule tissue 21 months after the start of treatment and the immunohistochemical staining of *Wolbachia* within adult *O. volvulus* tissues 21 months after the start of treatment, c) the histological assessment of the frequency of embryonic stages present within female *O. volvulus* uteri 21 months after the start of treatment, d) the detection of adult *O. volvulus* motility within onchocercomas using ultrasonography e) histological assessment of parasite viability, f) the clinical monitoring and assessment of adverse reactions during primary drug allocation (doxycycline) or secondary drug allocation (ivermectin) in patients singly infected with *O. volvulus* or co-infected with *L. loa* and g) the number of *L. loa* and *M. perstans* mf present in 50 µl thick blood smears taken at baseline, 4, 12 and 21 months after the start of treatment.

### Assessment of *O. volvulus* microfilaridermia

Two skin snip samples of approximately 1 mg were taken from the rear of the leg using a Walser skin punch. Skin snips were placed in 200 µl saline containing 2 mM EDTA and incubated overnight at room temperature. The following day the saline samples were mounted on glass slides, total numbers of released mf were counted using a compound microscope and the mean number of mf/snip was derived.

### Assessment of *L. loa* and *M. perstans* microfilaraemia

Finger prick blood samples (50 µl) were taken at baseline and 4, 12 and 21 month after the start of treatment. Thick blood smears were made to count numbers of *L. loa* and *M. perstans* mf by microscopy.

### Determination of *Wolbachia* levels, embryogenic status and viability

Onchocercomas were surgically removed under local anaesthesia from operable sites. Onchocercomas were halved and fixed in either 80% ethanol for histology or in stabilisation buffer (RNAlater, Qiagen) for DNA analysis. Genomic DNA was extracted and the quantity of *Wolbachia wsp* was determined by quantitative PCR as previously described [24]. Ethanol fixed tissue was embedded in paraffin wax blocks and several sections stained with haematoxylin and eosin. The embryonic status of the adult females was determined by counting the number of adult female cross sections which contained ova, morulae, curled microfilariae and straight microfilariae. Immunohistochemistry for the detection of *Wolbachia* used a polyclonal rabbit antisera raised to *B.malayi Wolbachia* surface protein (WSP) at a dilution of 1∶2000 [24] and for viability with a rabbit antisera to lysosomal aspartic protease of *O. volvulus* (APR) at a dilution of 1∶1000 [25]. The viability of parasites was assessed using criteria as previously described [19]. In brief, the criteria for dead worms included evidence of calcification without cuticle or nearly complete adsorbed, loss of body wall integrity, loss of nuclei and absence of APR staining.

### Determination of adult motility

Ultrasonography (USG) was used to examine palpable onchocercomas as previously described [26]. Ultrasound examinations were performed 21 months after treatment start using a portable ultrasound system (Sonosite 180 Plus®, Sonosite Washington, USA) and a linear transducer (L38mm) with frequencies of 7.5–10 MHz. Patients were examined in a supine position in order to avoid artefacts due to movements. Each onchocercoma was scanned in longitudinal and transverse sections to detect motile adult filariae. The transducer was positioned at the largest diameter or at the largest echo-free area in case of cystic nodules. Imaging was carried out in panorama mode to provide optimal information. The detection of all onchocercomata was recorded with a camcorder (SONY® PAL handycam, SONY Corp, Japan) on video tapes. Onchocercomata were identified by a capsule of connective tissue, lateral shadowing, partly echo-free areas as sign for necrotic proceedings and acoustic shadowing, reflecting moving and static fragments of the adult worms [26].

### Assessment of adverse events

Clinical monitoring of adverse reactions was undertaken by community health officers throughout the 6-week period of doxycycline treatment. Patients were asked by questionnaire for any side effects of the drugs as per protocol. Adverse events were assigned scores; 0 = no abnormality, 1 = mild, 2 = moderate and 3 = severe. Individuals were asked to report any signs and symptoms that were not experienced prior to drug administration. All symptoms were documented in patients' treatment cards and medication or hospitalisation was provided where necessary. For the assessment of adverse reactions to ivermectin, a scoring system previously described was utilized [27]. Incidence and severity of clinical symptoms consistent with ivermectin-associated adverse reactions (such as increased body temperature and type and extent of skin rash) were recorded immediately preceding ivermectin treatment and forty-eight hours following administration.

### Sample size

Based on data from a previous study [17], a reduction in the (geometric) mean mf load/mg skin of 50% at 6 months was considered to be clinically significant. Assuming a baseline (geometric) mean mf load of 9.40 and a standard deviation similar to that in the previous study, 25 patients would be sufficient to detect such a reduction with 80% power. To allow for up to a 15% drop-out rate over the study period, 30 patients were recruited into each treatment group.

### Randomization and blinding

Randomization for onchocerciasis was block stratified based on baseline microfilaridermia. All *L. loa* co-infected patients were assigned to doxycycline + ivermectin treatment. Treatment allocation was assigned by randomized ID code (by JDT and MJT) and the course of treatment sealed in an envelope for allocation by the field team and district field officers. All study personnel and participants were blinded to the doxycycline and placebo treatment assignment for the duration of the study.

### Deviation from protocol

Placebo tablets for assessment of ivermectin adverse events were not supplied in time for treatment allocation and so unmarked lactose tablets of similar size, shape and colour were used as an alternative. The ivermectin and dummy pills were assigned to individuals in sealed unmarked envelopes before being handed over to district health officers for drug delivery and these together with individuals responsible for the clinical assessment of adverse events were not involved in any subsequent analysis. Due to the lack of significant differences between groups in the severity or incidence of adverse reaction, cytokine analysis was not performed.

### Statistical analysis

The drop-out rates in the treatment groups were compared using Kaplan-Meier (survival curve) analyses. The age distributions and sex ratios of the groups were compared using one-way ANOVA and the Fisher exact test respectively. *O. volvulus* microfilaridermia and *wsp* copy number measurements were significantly positively skewed (even after log transformation) as assessed by the Kolmogorov–Smirnov test of Normality, so were evaluated using non-parametric analyses; changes in mf counts with time from baseline were assessed using Wilcoxon signed rank tests and differences in mf counts between the treatment groups were analysed using Mann-Whitney U tests. As group sizes were small, frequency of amicrofilaridermia, mf in onchocercomatous tissue, embryonic stages within uteri and adult worm movement were compared across the treatment groups using Fisher exact tests. All analyses were performed using the SPSS v11, Stata8 and GraphPad Prism software packages.

## Results

### Participant flow and recruitment

**Figure 1:**
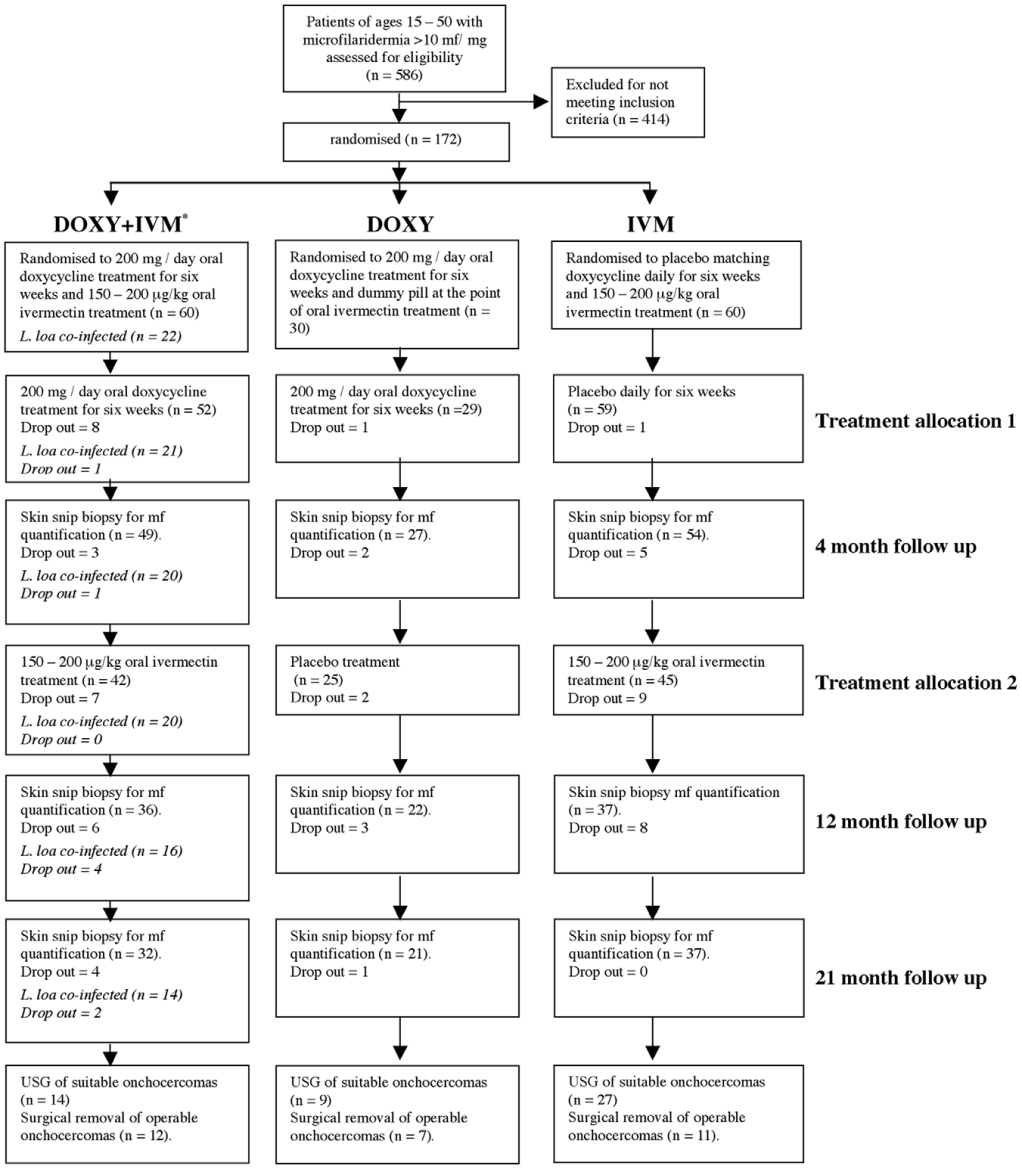
Trial profile. *All *L.loa* positive patients were additionally assigned to this group (numbers and dropouts provided in italics).

Figure 1 illustrates the trial profile. One hundred and fifty onchocerciasis patients fulfilling all entry criteria, were enrolled into the trial and randomized into one of the three treatment arms. Twenty-two additional onchocerciasis patients fulfilling all entry criteria were identified as positive for *L. loa* infection (below the safety threshold of 8000 mf/ml) and assigned into doxycycline + ivermectin regimen.

### Adherence to treatment and drop out

102/112 (91%) of the individuals who started treatment completed the full course of doxycycline and 59/60 (98%) placebo. For ivermectin 107/123 (87%) and dummy pill 25/27 (93%) individuals completed treatment. Additional drop-outs at the 4, 12 and 21 month follow-up assessments were 11, 21 and 7, respectively. Thus, in total, 104 out of 172 enrolled patients (62%) completed all treatment allocations and all subsequent follow-ups and were included for outcome analysis. There were no significant differences in the drop-out patterns over the follow up period between the three treatment groups (log-rank test of survival p = 0.718).

The baseline characteristics of these patients are reported in Table 1. Age and gender did not significantly differ between the three treatment groups.

**Table 1:** Characteristics of patient groups that completed the trial (enrolment, treatment allocations and all follow ups).

Infection group	Treatment	n	Mean age (range)^1^	Sex ratio: male/female^2^	Patients with palpable onchocercomas	Median *O. volvulus* microfilaridermia mf/snip (range)	Median *M. perstans* microfilaraemia mf/ml (range)	Median *L. loa* microfilaraemia mf/ml (range)
*O. volvulus*	Doxycycline+ Ivermectin	20	35.0 (15-50)	13 (65%)/7 (35%)	5 (25%)	24.25 (11.00-101.00)	–	–
*O. volvulus + M. perstans*	Doxycycline + Ivermectin	12	35.6 (20–50)	7 (58%)/5 (42%)	7 (58%)	27.50 (10.00–200.00)	40.00 (1.00–560.00)	–
*O. volvulus + L. loa +/− M. perstans*	Doxycycline + Ivermectin	14	32.1 (15–50)	11 (79%)/3 (21%)	8 (57%)	35.75 (9.50–145.00)	0.00 (0.00–700.00)	150.00 (20.00–5540.00)
*O. volvulus*	Doxycycline	21	34.8 (15–50)	13 (62%)/8 (38%)	14 (67%)	23.50 (9.5–124.50)	–	–
*O. volvulus*	Ivermectin	23	37.9 (22–50)	12 (52%)/11 (48%)	9 (52%)	32.00 (11.5–154.00)	–	–
*O. volvulus + M. perstans*	Ivermectin	14	34.9 (16–50)	6 (43%)/8 (57%)	7 (50)%	33.25 (11.5–102.50)	90.00 (1.00–160.00)	–

1 No significant difference in age distribution between treatment groups [one way ANOVA: F(2,101)  =  0.3346, p  =  0.7164].

2 No significant difference in sex ratios between treatment groups:

(a) Doxycycline + ivermectin (*O. volvulus* single infection) and doxycycline + ivermectin (*L. loa* co-infected) separate:*Fisher exact test ns.*

(b) Doxycycline + ivermectin (*O. volvulus* single infection) and doxycycline + ivermectin (*L. loa* co-infected) combined: *Fisher exact test ns.*

### Outcome analyses

For the assessment of adverse events, patients co-infected with *L. loa* (treated with doxycycline and ivermectin) have been analysed as a distinct group in order to evaluate whether co-infection is associated with the occurrence of such events. For all other outcome analyses, these patients have been combined with *O. volvulus* single infected patients receiving doxycycline and ivermectin.

### Occurrence of adverse events during treatment allocations

Table 2 summarizes the recorded adverse events during the 6-week primary drug allocation of doxycycline or matching placebo and 48 hours following the secondary drug allocation of ivermectin or dummy pill allocation. Adverse events were recorded in 17 patients during the 6-week period of doxycycline or matching placebo allocation. The incidence of adverse event did not significantly deviate between doxycycline- or placebo-assigned patients or between *O. volvulus* single infected and *O. volvulus* + *L. loa* co-infected patients assigned doxycycline. Symptoms were mild and included itching, fever, headache, body pains and vertigo. One patient administered doxycycline developed fever and headache, which led to a temporary interruption of the treatment for 5 days. Anti-malarial drugs were given for three days and doxycycline treatment resumed after recovery. There was no evidence or complaint of symptoms consistent with doxycycline-associated photosensitivity. It was not necessary to discontinue primary drug allocation in any instance.

**Table 2:** incidence and severity of adverse events during doxycycline and following ivermectin treatment.

Patient Group	n	Incidence of adverse event during 6 week doxycycline treatment^1^	n	Incidence of adverse reaction 48 h following ivermectin treatment^2^			
				None	Mild	Moderate^2^	Severe
Doxycycline + Ivermectin (*O. volvulus* single infection)	52	6 (11.5%)	42	39 (92.9%)	3 (7.1%)	0	0
Doxycycline + Ivermectin (*O. volvulus* + *L. loa* co-infection)	21	2 (9.5%)	20	17 (85.0%)	3 (15.0%)	0	0
Doxycycline	29	2 (6.9%)	25	22 (88.0%)	3 (12.0%)	0	0
Ivermectin	59	7 (11.9%)	45	38 (84.4%)	5 (11.1%)	2 (4.4%)	0

1 No significant differences between the four treatment groups with respect to the frequency of adverse event during 6 week doxycycline treatment. (Fisher exact test: p = 0.964 n.s.).

2 No significant difference in frequencies of adverse reactions between treatment groups: Doxycycline + ivermectin (*O. volvulus* single infection) and doxycycline + ivermectin (*L. loa* co-infected) combined: *Fisher exact test p = 0.538 ns.*

Forty-eight hours following ivermectin or dummy pill allocation, symptoms consistent with adverse reactions were observed in 16 patients (12.1% of all patients present). The majority of the adverse reactions were graded as mild (14 patients) with two patients in the ivermectin only group experiencing moderate adverse reactions. The frequency of either mild or moderate adverse reaction did not significantly differ between patients treated with ivermectin following doxycycline intervention and patients treated with ivermectin following placebo, matching doxycycline. Furthermore, the frequency of adverse reaction did not differ between patients treated with ivermectin and patients treated with a dummy pill.

### Reductions in *O. volvulus* mf

**Figure 2:**
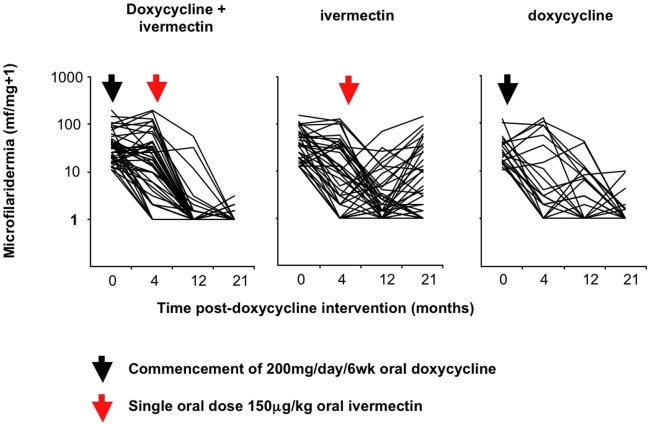
Changes in *Onchocerca volvulus* microfilaridermia from baseline at 4 months, 12 months and 21 months post treatment with doxycycline + ivermectin, doxycycline only or ivermectin only. Arrows indicate commencement of intervention (black arrow; doxycycline, red arrow; ivermectin).

Figure 2 illustrates the changes in *O. volvulus* mf levels in skin from baseline. The analysis of these changes is summarized in Table 3. Baseline, *O. volvulus* microfilaridermia did not significantly differ between treatment groups in the participants who completed the trial. At 4 months post-doxycycline allocation and immediately preceding ivermectin allocation, reductions in microfilaridermia had occurred in all treatment groups compared with baseline; these were statistically significant both for the doxycycline and placebo groups assigned for ivermectin allocation but not for the group assigned to doxycycline alone. No significant inter-treatment group differences in microfilaridermia were observable at 4 months. At 12 months post-doxycycline intervention (8 months post-ivermectin intervention), microfilaridermia was significantly reduced in all treatment groups. However, inter-treatment group differences were also apparent at this follow up. Doxycycline + ivermectin treated individuals had lower levels of microfilaridermia compared with both the ivermectin only and doxycycline only groups. Also, the incidence of amicrofilaridermia (an absence of detectable mf in the skin) was significantly higher in doxycycline + ivermectin groups (76.1%) compared with both the ivermectin only (21.6%) and doxycycline only (38.1%) groups. At 21 months post-doxycycline intervention (17 months post-ivermectin intervention), significant microfilaridermia reductions from baseline persisted in all treatment groups. Inter-treatment differences between doxycycline + ivermectin and ivermectin groups were also preserved. Furthermore, the majority (89.1%) of the doxycycline + ivermectin treatment group were amicrofilaridermic at 21 months, compared with 21.6% in the ivermectin only treatment group. At 21 months the doxycycline only group also showed greater reductions in microfilaridermia and increased frequency of amicrofilaridermia (66.7%) compared with ivermectin only. When comparing between doxycycline + ivermectin and doxycycline only at 21 months, the combined treatment group showed significantly increased frequency of amicrofilaridermia.

**Table 3:** Changes in *O. volvulus* microfilaridermia at 4, 12 and 21 months after the start of treatment.

Treatment	n	Baseline median microfilaridermia mf/snip (range)	4 month median microfilaridermia mf/snip (range) *No. of participants with amicrofilaridermia (%)*	12 month median microfilaridermia mf/snip (range) *No. of participants with amicrofilaridermia (%)*	21 month median microfilaridermia mf/snip (range) *No. of participants with amicrofilaridermia (%)*
Doxycycline + Ivermectin	46	31 (10–200)	16 (0–179) *6 (13.0%)*	0 (0–54.5) *35 (76.1%)*	0 (0–2) *41 (89.1%)*
Doxycycline	21	23.5 (11–124.5)	2 (0–186) *5 (23.8%)*	2 (0–57.5) *8 (38.1%)*	0 (0–8.5) *14 (66.7%)*
Placebo/Ivermectin	37	32 (12–154)	8 (0–121) *7 (18.9%)*	1.5 (0–69) *8 (21.6%)*	4 (0–141) *8 (21.6%)*

Analysis of longitudinal changes in microfilaridermia from baseline per treatment group (Wilcoxon Signed Rank tests):

Doxycycline + ivermectin: p = 0.0295 (^*^ 4 month); p<0.0001 (^***^12 month); p<0.0001 (^***^ 21 month)\

Doxycycline: p = 0.2760 (ns, 4 month); p = 0.0020 (^**^ 12 month); p<0.0001 (^***^ 21 month).

Ivermectin: p = 0.0090 (^***^4 month); p<0.0001 (^***^12 month); p = 0.0005 (^***^ 21 month).

Analysis of differences in microfilaridermia between treatment groups at baseline, 4, 12 and 21 months post treatment (Mann Whitney U tests):

Doxycycline + ivermectin vs ivermectin: p = 0.7381 (ns, baseline); p = 0.2335 (ns, 4 month); p<0.0001 (^***^ 12 month); p<0.0001 (^***^ 21 month).

Doxycycline vs ivermectin: p = 0.5712 (ns, baseline); p = 0.2335 (ns, 4 month); p = 0.7214 (ns, 12 month); p = 0.0002 (^***^ 21 month).

Doxycycline + ivermectin vs doxycycline: p = 0.5841 (ns, baseline); p = 0.1990 (ns, 4 month); p = 0.0025 (^***^ 12 month); p = 0.1193 (ns, 21 month).

Analysis of amicrofilaridermia frequencies between treatment groups at 4, 12 and 21 months post treatment (Fisher exact tests):

Doxycycline + ivermectin vs ivermectin: p = 0.5417 (ns, 4 month); p<0.0001 (^***^ 12 month); p<0.0001 (^***^ 21month).

Doxycycline vs ivermectin: p = 0.7409 (ns, 4 month); p = 0.2265 (ns, 12 month); p = 0.0016 (^***^ 21month).

Doxycycline + ivermectin vs doxycycline: p = 0.3006 (ns, 4 month); p = 0.0052 (^**^ 12 month); p = 0.0394 (^*^ 21month).

### Depletion of *Wolbachia* from adult *O. volvulus*

**Figure 3:**
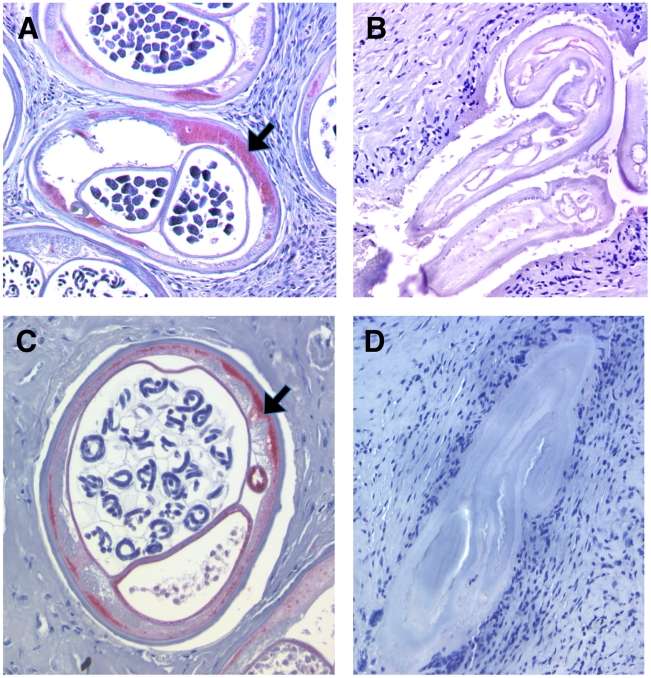
Immunohistochemical assessment of *Wolbachia* and nematode viability within adult *O. volvulus* tissue derived from trial patients at 21 months after the start of treatment. A: WSP-specific immunostaining (arrow) of *Wolbachia* in *O. volvulus* adult female after ivermectin only treatment, B: Lack of WSP staining in *O. volvulus* from a doxycycline + ivermectin treated individual. C: APR staining (arrow) of viable adult female *O. volvulus* after ivermectin only treatment. D: Lack of APR staining in a non-viable adult worm from a doxycycline treated individual.

Table 4 summarizes the measurements of *Wolbachia* within adult *O. volvulus* derived from extirpated nodules at 21 months. In total 30 individuals who completed the trial were selected for nodulectomy (selection based on the presence of palpable nodules in suitable sites for operation). *Wolbachia wsp* copy number, determined from genomic DNA extracted from one half of each extirpated nodule, was significantly lower in both doxycycline + ivermectin and doxycycline only treatment groups, compared with ivermectin only treatment. The depletion of *Wolbachia* in doxycycline treatment groups determined by PCR was corroborated by immunohistochemical staining of *Wolbachia* WSP within sections of adult *O. volvulus* tissues (Table 4, Figure 3). In both doxycycline only and doxycycline + ivermectin treated individuals, WSP positive staining was not detected in any of the nodules examined. These frequencies were significantly lower when compared with the frequency of positive staining of *O. volvulus* tissues derived from patients treated with ivermectin only (67%).

**Table 4:** Changes in *Wolbachia* surface protein expression and *wsp* DNA copy number in onchocercomas 21 months following start of treatment.

Treatment	Nodulectomy patients (total number of onchocercomas)	WSP % +ve^1^ (no. +ve sections/no. total sections)	Median *wsp* copy number^2^ (range)
Doxycycline + Ivermectin	12 (14)	0^**^ (0/14)	0^*^ (0–15389)
Doxycycline	7 (12)	0^**^ (0/11)	0^*^ (0–800)
Ivermectin	11 (29)	66.7 (14/21)	484 (0–99805)

1 Analysis of the frequency of *O. volvulus* tissue positively stained with anti-WSP antibody between treatment groups at 21 months post treatment (Fisher exact tests):

Doxycycline + ivermectin vs ivermectin: p = 0 005 (^**^).

Doxycycline vs ivermectin: p = 0.003 (^**^).

Doxycycline + ivermectin vs doxycycline: p = 1.000 (ns).

2 Analysis of differences in *wsp* DNA copy number within onchocercomas between treatment groups at 21 months post treatment (Mann-Whitney U-tests):

Doxycycline + ivermectin vs ivermectin: p = 0.015 (^*^).

Doxycycline vs ivermectin: p = 0.013 (^*^).

Doxycycline + ivermectin vs doxycycline: p = 0.752 (ns).

### Reductions in *O. volvulus* embryogenesis

Histological observations of different embryonic stages within uteri and released mf in nodule tissue are summarized in Table 5. The frequency of ova, morulae, curled stage and straight stage embryos within uteri were reduced in female worms derived from doxycycline + ivermectin treated individuals compared with ivermectin only treated individuals after 21 months. Similar reductions in frequency of embryonic developmental stages were observed in doxycycline only treated individuals compared with ivermectin only treated individuals. Presence of mf within nodule tissue sections were also reduced in both doxycycline + ivermectin and doxycycline only treated individuals compared with ivermectin only treated individuals at 21 months.

**Table 5:** Histological analysis of embryonic stages within uteri and released mf in onchocercoma tissue.

Treatment	Nodulectomy patients (total number of onchocercomas)	Uterine contents % +ve (no. +ve cross sections/no. total cross sections)				Released mf^5^ % +ve (no. +ve sections/no. total sections)
		Ova^1^	Morulae^2^	Curled mf^3^	Straight mf^4^	
Doxycycline + Ivermectin	12 (14)	4.0^***^ (22/546)	2.9^***^ (16/546)	3.3^***^ (18/546)	0.9^***^ (5/546)	19.4^*^ (6/31)
Doxycycline	7 (12)	4.1^***^ (20/492)	2.0^***^ (10/492)	2.0^***^ (10/492)	1.0^***^ (5/492)	7.4^***^ (2/27)
Ivermectin	11 (29)	9.0 (123/1363)	9.0 (123/1363)	17.0 (232/1363)	8.0 (109/1363)	47.1 (24/51)

All of the following analyses were performed using Fisher exact tests:

1 Analysis of the frequency of ova present within uterine cross sections of female *O. volvulus* between treatment groups at 21 months post treatment:

Doxycycline + ivermectin vs ivermectin: p<0.001 (^***^).

Doxycycline vs ivermectin: p<0.001 (^***^).

Doxycycline + ivermectin vs doxycycline: p = 1.000 (ns).

2 Analysis of the frequency of morulae present within uterine cross sections of female *O. volvulus* between treatment groups at 21 months post treatment:

Doxycycline + ivermectin vs ivermectin: p<0.001 (^***^).

Doxycycline vs ivermectin: p<0.001 (^***^).

Doxycycline + ivermectin vs doxycycline: p = 0.428 (ns).

3 Analysis of the frequency of curled mf present within uterine cross sections of female *O. volvulus* between treatment groups at 21 months post treatment:

Doxycycline + ivermectin vs ivermectin: p<0.001 (^***^).

Doxycycline vs ivermectin: p<0.001 (^***^).

Doxycycline + ivermectin vs doxycycline: p = 0.251 (ns).

4 Analysis of the frequency of straight mf present within uterine cross sections of female *O. volvulus* between treatment groups at 21 months post treatment:

Doxycycline + ivermectin vs ivermectin: p<0.001 (^***^).

Doxycycline vs ivermectin: p<0.001 (^***^).

Doxycycline + ivermectin vs doxycycline: p = 1.000 (ns).

5 Analysis of the frequency of released mf present within onchocercoma tissue between treatment groups at 21 months post treatment:

Doxycycline + ivermectin vs ivermectin: p = 0.017 (^*^).

Doxycycline vs ivermectin: p<0.001 (^***^).

Doxycycline + ivermectin vs doxycycline: p = 0.263 (ns).

### Reductions in adult *O. volvulus* worm motility

Table 6 documents the results of USG examination. At the 21-month follow up, 50 patients with palpable nodules undertook ultrasound examination. Distinct adult worm movement could be observed within nodules in 44.0% of ivermectin treated patients. In comparison, the frequencies of detectable worm movement in doxycycline + ivermectin (7.1%) or doxycycline only (11.1%) treated individuals was significantly lower only for the doxycycline + ivermectin group.

**Table 6:** Analysis of USG detection of adult *O. volvulus* worm movement 21 months after the start of treatment.

Treatment	USG patients	% +ve detectable adult movement^1^ (no. +ve/total no.)
Doxycycline + Ivermectin	14	1/14 (7.1%)^*^
Doxycycline	9	1/9 (11.1%)
Ivermectin	27	12/27 (44.0%)

1 Analysis of the frequency of detectable adult *O. volvulus* movement within onchocercomas between treatment groups at 21 months post treatment (Fisher exact tests): Doxycycline + ivermectin vs ivermectin: p = 0.031 (^*^). Doxycycline vs ivermectin: p = 0.114 (ns). Doxycycline + ivermectin vs doxycycline: p = 1.000 (ns).

### Reduction in *O.volvulus* adult worm viability

At 21 months the proportion of dead adult female worms from patients in the doxycycline + ivermectin (47%) and doxycycline only groups (65%) was significantly increased compared to the ivermectin only group (17%). There was also a significant reduction in the number of living adult female and male worms per patient (Table 7).

**Table 7:** *O. volvulus* viability 21 months after the start of treatment.

Treatment	Patients	Nodules	Female worms^1^		Male worms^8^		Living worms per patient	
			all	dead	all	dead	Female^4^	Male^9^
	n	n	n	n (%)	n	n (%)	mean (se)	mean (se)
Doxycycline + ivermectin	6	9	15	7 (46.7)^2^	3	0	1.33 (0.42)^5^	0.50 (0.34)^10^
Doxycycline	7	9	20	13 (65.0)^3^	3	0	1.00 (0.44)^6,7^	0.43 (0.20)^11,12^
Ivermectin	13	27	54	9 (16.7)	22	1 (4.5)	3.46 (0.56)	1.62 (0.43)

Female worms.

1 Significant differences in proportions of dead worms between the three groups (Fishers exact test: p<0.001).

2 Significant difference in proportion of dead female worms compared to ivermectin only group (Fishers exact test: p = 0.033).

3 Significant difference in the proportion of dead female worms compared to ivermectin only group (Fishers exact test: p<0.001).

4 Assuming a Poisson distribution: significant differences in mean numbers of living worms per patient between the three groups (χ^2^(2) = 16.01, p<0.001.

5 placebo + ivermectin vs. doxycycline + ivermectin p = 0.006.

6 placebo + ivermectin vs. doxycycline p<0.001.

7 doxycycline + ivermectin vs. doxycycline p = 0.578 (ns).

Male worms.

8 No significant differences in proportions of dead worms between the three groups (Fishers exact test: p = 1.000).

9 Assuming a Poisson distribution: significant differences in mean numbers of living worms per patient between the three groups (χ^2^(2) = 8.86, p = 0.012.

10 placebo + ivermectin vs. doxycycline + ivermectin p = 0.029.

11 placebo + ivermectin vs. doxycycline p = 0.012.

12 doxycycline + ivermectin vs. doxycycline p = 0.850 (ns).

### Changes in *L. loa* and *M. perstans* circulating mf

**Figure 4:**
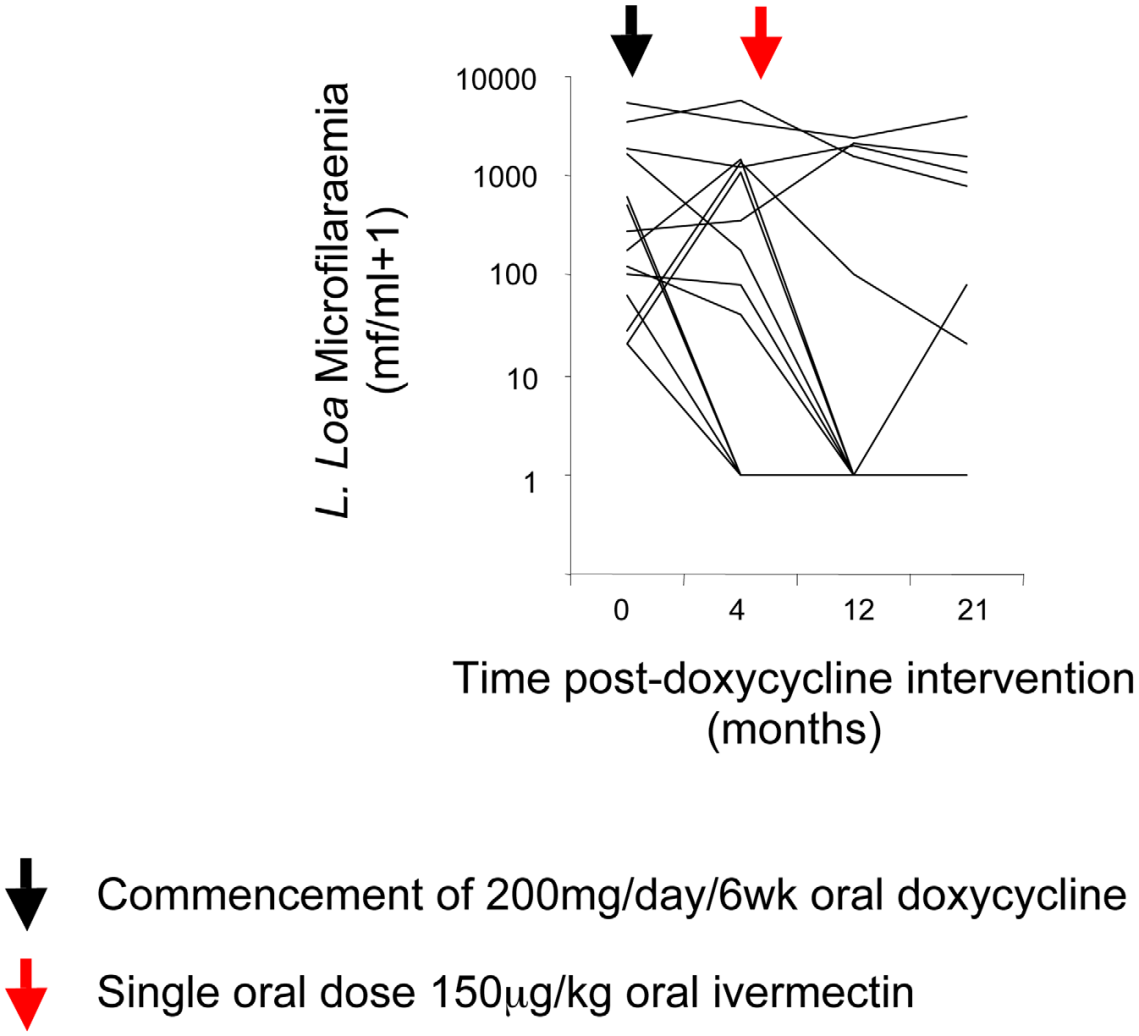
Changes in *Loa loa* microfilaridermia from baseline at 4 months, 12 months and 21 months post treatment with doxycycline + ivermectin, doxycycline only or ivermectin only. Arrows indicate commencement of intervention (black arrow; doxycycline, red arrow; ivermectin).

Fluctuations in *L. loa* microfilaraemia from baseline are illustrated in Figure 4 and statistical differences summarized in Table 8. The study design precluded a treatment comparison (all *L. loa* patients were assigned doxycycline + ivermectin) and therefore only longitudinal analyses were undertaken. No significant changes in *L. loa* microfilaraemia occurred at 4 months after doxycycline treatment (immediately preceding ivermectin intervention). *L. loa* mf loads were significantly reduced at 12 months and 21 months after the start of doxycycline treatment (8 and 17 months after ivermectin treatment).

**Table 8:** Changes in *L. loa* parasitaemia at 4, 12 and 21 months after the start of treatment.

Treatment	n	Baseline median parasitaemia mf/ml (range)	4 month median parasitaemia mf/ml (range) *No. of participants with amicrofilaraemia (%)*	12 month median parasitaemia mf/ml (range) *No. of participants with amicrofilaraemia (%)*	21 month median parasitaemia mf/ml (range) *No. of participants with amicrofilaraemia (%)*
Doxycycline + Ivermectin	14	150	130	0^*^	0^**^
		(20–5540)	(0–5820)	(0–2440)	(0–3840)
			*6 (37.5%)*	*11 (68.8%)*	*10 (62.5%)*

Analysis of longitudinal changes in *L. loa* microfilaraemia from baseline in patients treated with Doxycycline + ivermectin (Wilcoxon Signed Rank tests): 4 month: (p = 0.623, ns); 12 month: p = 0.049 (^*^); 21 month: p = 0.010 (^**^).

**Figure 5:**
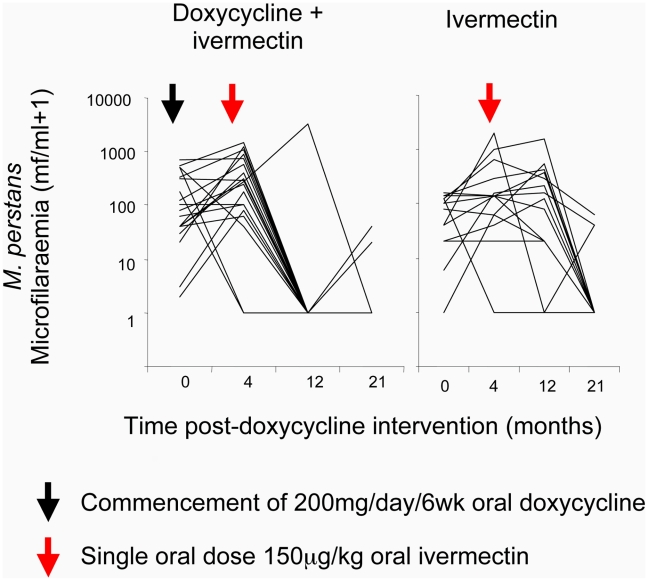
Changes in *Mansonella perstans* microfilaridermia from baseline at 4 months, 12 months and 21 months post treatment with doxycycline + ivermectin, doxycycline only or ivermectin only. Arrows indicate commencement of intervention (black arrow; doxycycline, red arrow; ivermectin).

*M. perstans* microfilaraemia had significantly increased from baseline at 4 months after doxycycline allocation, immediately preceding ivermectin intervention, in both doxycycline and placebo treatment groups (Table 9, Figure 5). No inter-group differences in *M. perstans* mf levels at 4 months were observable. At 12 months after the start of doxycycline intervention (8 months following ivermectin intervention) significant reductions in circulating mf compared with baseline levels had occurred in doxycycline + ivermectin treated individuals but not ivermectin only treated individuals. There was also a strong inter-group difference at 12 months in both *M. perstans* microfilaraemia and frequency of amicrofilaraemia (94.7% in doxycycline + ivermectin compared with 7.1% in ivermectin only treated individuals). By the 21-month follow up (17 months following ivermectin treatment), both treatment groups showed reductions in *M. perstans* microfilaraemia compared with baseline. There were no inter-group differences apparent at this stage with amicrofilaraemia occurring in the majority of both doxycycline + ivermectin (84.2%) and ivermectin only (78.6%) treated individuals.

**Table 9:** Changes in *M. perstans* parasitaemia at 4, 12 and 21 months after the start of treatment.

Treatment	n	Baseline median parasitaemia mf/ml (range)	4 month median parasitaemia mf/ml (range)	12 month median parasitaemia mf/ml (range)	21 month parasitaemia mf/ml (range)
			*No. of participants with amicrofilaraemia (%)*	*No. of participants with amicrofilaraemia (%)*	*No. of participants with amicrofilaraemia (%)*
Doxycycline + Ivermectin	19	80	280	0^***^	0
		(1–700)	(0–1460)	(0–3200)	(0–40)
			*0*	*18 (94.7%)*	*16 (84.2%)*
Ivermectin	14	90	140	190	0
		(1–160)	(0–1940)	(0–1540)	(0–60)
			*1 (7.1%)*	*1 (7.1%)*	*11 (78.6%)*

Analysis of longitudinal changes in *M. perstans* microfilaraemia from baseline (Wilcoxon Signed Rank tests):

Doxycycline + ivermectin: p = 0.033 (^*^ 4 month); p = 0.002 (^**^ 12 month); p = 0.015 (^*^ 21 month).

Ivermectin: p = 0.028 (^*^ 4 month); p = 0.069 (ns, 12 month); p = 0.001 (^***^ 21 month).

Analysis of differences in *M. perstans* microfilaraemia between treatment groups at baseline, 4, 12 and 21 months post treatment (Mann Whitney U tests):

Doxycycline + ivermectin vs ivermectin: p = 0.418 (ns, baseline); p = 0.358 (ns, 4 month); p<0.001 (^***^ 12 month); p = 0.843 (ns, 21 month).

Analysis of frequency *M. perstans* amicrofilaraemia between treatment groups at 12 and 21 months post treatment (Fisher exact tests):

Doxycycline + ivermectin vs ivermectin: p = 0.424 (ns, 4 months); p<0.001 (^***^12 month); p = 1.000 (ns, 21 month).

## Discussion

The major outcome of this trial is that a 6-week course of doxycycline alone is a highly effective treatment against onchocerciasis, leading to long term and profound suppression of microfilaridermia, embryogenesis and significant macrofilaricidal activity independently of ivermectin administration. It also shows that an additional treatment with ivermectin does not lead to any improvement in the macrofilaricidal or sterilising activity of doxycycline. Furthermore, our trial indicates that doxycycline treatment is well-tolerated in *O. volvulus* patients co-infected with low to moderate levels of *L. loa* parasitaemias. These findings promote the use of a doxycycline only regimen to treat onchocerciasis patients co-infected with *L. loa*. However, further trials are warranted to test safety and efficacy of doxycycline treatment in co-infected individuals at risk of developing serious adverse reactions to ivermectin. Should the results of such trials support our safety and efficacy findings reported here, a potential solution will be available to MDA programs currently disrupted by the threat of *L. loa* SAE. Previous trials on lymphatic filariasis that also demonstrated macrofilaricidal activity with doxycycline alone [28] suggest that this approach could be extended to co-infections of *Wuchereria bancrofti* and loiasis.

The results of this trial confirms previous findings in onchocerciasis and lymphatic filariasis that a course of doxycycline sufficient to deplete *Wolbachia* by >90% results in the death of adult worms (reviewed in [29]). Prior to the removal of the nodules for histochemical and PCR analysis we used ultrasonography (USG) to detect the *in vivo* motility of parasites. The USG data showed reduced parasite motility in doxycycline + ivermectin treated individuals compared with the ivermectin only group and suggests that USG maybe used as a non-invasive tool to assess potential macrofilaricidal activity prior to histological analysis of parasite viability. Histological and PCR analysis confirmed that doxycycline treatment resulted in loss of *Wolbachia* from the adult parasites, with an extensive loss of uterine contents reflecting a blockage of embryogenesis as previously observed [18], [19].

Treatment with doxycycline + ivermectin or doxycycline alone is superior to ivermectin in achieving sustained reductions in skin mf. The kinetics of mf decline are in line with the different modes of actions of the two drugs. The slow decline in mf skin levels following doxycycline treatment is most likely a consequence of the block in embryogenesis preventing the release of mf into the skin. These kinetics are beneficial in avoiding the rapid death of mf, which in individuals with high parasite burden leads to inflammatory Mazzotti adverse events following anti-filarial drug treatment and are associated with the release of *Wolbachia* into the blood and tissues [30], [31]. The low incidence of adverse events following ivermectin treatment in this trial probably reflect the relatively low *O. volvulus* microfilarial burden, which was further reduced by a reduction in *O. volvulus* microfilaridermia levels across all groups from baseline to the 4 month follow-up time point (prior to ivermectin administration). The absence of SAE in *L. loa* co-infected individuals was as expected due to exclusion of patients with >8000 mf/ml parasitaemias. The tolerability of treatment with doxycycline is consistent with our previous experience in more than 1000 treated field trial volunteers. No experience of severe adverse event or evidence of photosensitivity has been recorded. For ethical and safety issues, our study design precluded enrolment of individuals co-infected with *L. loa* above the safety threshold for standard ivermectin treatment (>8000 mf/ml). However, we noted no additional safety issues in *L. loa* co-infected patients during doxycycline allocation. The dropout rate for the trial did not differ between *L. loa* positive and negative treatment groups. Given that our trial demonstrates comparable efficacies of doxycycline with or without ivermectin, we believe further trials are warranted to determine the safety of doxycycline treatments in *L. loa* patients with >8000 mf/ml with *O. volvulus* co-infection. In this regard a trial of community directed delivery of doxycycline in an area of onchocerciasis and loiasis co-endemicity has been completed and demonstrates the feasibility of the use of large-scale doxycycline therapy for the control of onchocerciasis in such communities [38]. Some of the communities covered by this trial included those with a prevalence of loiasis in excess of 40%, where it can be estimated that more than 5% of this population would have microfilaraemia levels above the threshold of 8000 mf/ml. The lack of any SAE to doxycycline therapy in the 12,612 people that completed the course of treatment provides indirect evidence that doxycycline therapy appears safe in individuals with higher *L. loa* microfilaraemia.

Although the results of this trial show no additional benefit of ivermectin to the macrofilaricidal and sterilising activity of doxycycline, the combination of both drugs improves reductions in *O. volvulus* mf intensity and frequency of amicrofilaridermia. The timing of the ivermectin treatment may be sub-optimal and may be different in populations exposed to ivermectin control rather than the ivermectin-naïve population treated here.

The changes in *L. loa* microfilaraemia observed showed no change from baseline at the 4-month follow-up point. After ivermectin treatment mf loads were significantly reduced at both 12 and 21 month after the start of treatment as anticipated. The changes observed in *M. perstans* microfilaraemia showed a different pattern. At the 4-month follow-up mf levels had increased in both doxycycline and placebo groups and no differences between groups was observed. 8 months after ivermectin treatment there was a striking reduction in mf loads in the doxycycline group with no change from baseline levels observed in the ivermectin only group. At the 21-month follow up both groups showed marked reductions from baseline and high frequency of amicrofilaraemia. The difference between doxycycline and ivermectin only groups at the 12-month follow up would be consistent with a recent report that *M. perstans* is host to *Wolbachia* endosymbionts and a 6-week course of doxycycline leads to depletion of *Wolbachia* and microfilaraemia [32]. Although we were unable to confirm the presence of *Wolbachia* in *M. perstans* due to technical reasons, the observations of the changes to microfilaraemia at the 12-month follow up would be consistent with the presence and dependency of *Wolbachia* in *M. perstans.*

If the elimination of onchocerciasis and lymphatic filariasis (*W. bancrofti*) as a public health problem is to be achieved in Africa, a solution to the problem of *L. loa* co-endemicity in Central Africa has to be found. Attempts to use regimes of low dose ivermectin have failed to provide sufficient reductions in mf suitable for MDP and are probably inadequate to prevent the occurrence of post-treatment neurological SAEs [33]. Further trials are currently underway with albendazole, which can reduce *L. loa* microfilaraemia following a twice-daily 21-day course [34]. Shorter 3-day treatments with albendazole failed to lead to sufficient reductions safe enough for ivermectin treatment [35], [36] and so alternative regimes are required.

One such regime, which we have demonstrated in this trial, is the targeting of onchocerciasis with anti-wolbachial therapy. Current regimes with doxycycline are restricted for widespread MDA due to contraindications in children under 8 years old and pregnancy and the logistics of 4–6 week courses of treatment. Trials to evaluate the minimum effective course of treatment with combinations of doxycycline and rifampicin in onchocerciasis is currently underway as part of the A-WOL drug discovery and development programme, which aims to optimise current anti-wolbachial drugs and discover new drugs with a more rapid efficacy and without the contra-indications of doxycycline [37], (A-WOL.com). In addition, community directed intervention (CDI) trials using a 6-week course of doxycycline have been completed and challenge the notion that prolonged courses of treatment cannot be effectively delivered through CDI [38]. It may be possible to use RAPLOA, a rapid diagnostic tool, to map areas of high risk of encephalopathy to define restricted areas where these regimes could be deployed. Such regimes can already be considered as a suitable treatment for individual cases of onchocerciasis or LF in patients co-infected with *L. loa* and the further development of anti-wolbachial regimes compatible with MDA could offer an alternative tool for the control on onchocerciasis and LF in Africa.

## Supporting Information

Checklist S1 CONSORT checklist.
(0.04 MB DOC)
